# Characterizing diseases using genetic and clinical variables: A data analytics approach

**DOI:** 10.1002/qub2.46

**Published:** 2024-05-15

**Authors:** Madhuri Gollapalli, Harsh Anand, Satish Mahadevan Srinivasan

**Affiliations:** ^1^ Engineering Department Penn State Great Valley Malvern Pennsylvania USA; ^2^ Department of Systems and Information Engineering School of Engineering and Applied Science University of Virginia Charlottesville Virginia USA

**Keywords:** clustering, *k*‐means, L1000 dataset analysis, landmark genes, multinomial logistic regression, non‐landmark genes, principal component analysis, tissue classification

## Abstract

Predictive analytics is crucial in precision medicine for personalized patient care. To aid in precision medicine, this study identifies a subset of genetic and clinical variables that can serve as predictors for classifying diseased tissues/disease types. To achieve this, experiments were performed on diseased tissues obtained from the L1000 dataset to assess differences in the functionality and predictive capabilities of genetic and clinical variables. In this study, the *k*‐means technique was used for clustering the diseased tissue types, and the multinomial logistic regression (MLR) technique was applied for classifying the diseased tissue types. Dimensionality reduction techniques including principal component analysis and Boruta are used extensively to reduce the dimensionality of genetic and clinical variables. The results showed that landmark genes performed slightly better in clustering diseased tissue types compared to any random set of 978 non‐landmark genes, and the difference is statistically significant. Furthermore, it was evident that both clinical and genetic variables were important in predicting the diseased tissue types. The top three clinical predictors for predicting diseased tissue types were identified as morphology, gender, and age of diagnosis. Additionally, this study explored the possibility of using the latent representations of the clusters of landmark and non‐landmark genes as predictors for an MLR classifier. The classification models built using MLR revealed that landmark genes can serve as a subset of genetic variables and/or as a proxy for clinical variables. This study concludes that combining predictive analytics with dimensionality reduction effectively identifies key predictors in precision medicine, enhancing diagnostic accuracy.

## 1 INTRODUCTION

A cell interprets its genetic code in different ways and the expression of the genes enables the cell to control its size, shape, and functions. It is believed that gene expression affects an organism’s constitution/phenotype [1]. The Human Genome Project was started in 1988 as an international, collaborative research program to understand the complete mapping and functionality of the human genome [2]. The project included around 2800 researchers and the full sequence was completed and published in April 2003, providing a blueprint for building every human cell. While sequencing the genes tells us what the cell could possibly do, the gene expression profile tells us what it is doing at any given point in time [3]. It characterizes cellular states in response to disease, mutations, etc., and helps us understand how genes contribute to a specific disease or react to a specific treatment. Gene expression profiling, therefore, leads to uncovering key diagnostic tools for people with cancer. The ability to investigate the human genome has paved the way to understanding the association between genetic variation and complex diseases such as cardiovascular disease, cancer, diabetes, etc., and the treatments and preventive measures against these diseases are evolving [4]. There have been extensive studies to identify a particular disease risk group and better manage the treatment [5, 6]. The privilege to collect large data because of the emergence of new medical technologies further supports the advances in data analytics. The growing field of machine learning (ML) and artificial intelligence helps in the early prediction/diagnosis of carcinoma diseases including cancer susceptibility, recurrence, and survival prediction [7]. This study contributes to the efforts being carried out to develop data analytics techniques to assess the gene expression data and clinical outcome data from the National Cancer Institute (NCI) database. Precision medicine has been proven increasingly successful through clinical trials. The most important step in increasing patient survival rates is the early detection of disease.

Precision medicine, or personalized medicine, benefits when both the genomic and the clinical variables can be leveraged. The interaction between genetic variables and clinical variables such as the tumor stage, gender, and age at diagnosis results in a specific disease type, and any mutations in genes may result in an aggressive form of the disease. Several common cancer types share common genes. Though the genes *BRCA1* and *BRCA2* act as protective genes for a few types of cancers, any inherited mutations in them increase the likelihood of cancers such as breast, pancreatic, prostate, ovarian, and a few others [8]. Three percent of breast cancers and 10 percent of ovarian cancers per year are caused by mutations in *BRCA1* and *BRCA2* but they can also be caused by mutations inherited in other genes. Studies have reported success in clinical trials of precision medicine.

Clayman et al. used RNA‐Seq data along with clinical data to build models for the early detection of cancer that eventually increase the survival rates of cancer patients. Their models effectively predicted the overall survival of prostate cancer patients [7, 9]. Al‐Azzam and Shatnawi conducted a comparison of the supervised and semi‐supervised learning algorithms in a breast cancer prediction study. A total of nine classifiers including logistic regression, Gaussian Naïve Bayes, and linear support vector machine (SVM) were studied. Overall, the supervised learning algorithms outperformed the semi‐supervised techniques with the logistic regression reporting the best accuracy of 98%. However, the semi‐supervised classifiers reported a range of accuracies between 90% and 98% while using only half the training data [10]. In another study that classified triple‐negative breast cancer tissues, classification models of the RNA‐Seq data using SVM, Naïve Bayes, k‐nearest neighbor, and decision trees were explored. The SVM algorithm had the least misclassification errors of all [11]. In a study that analyzed breast cancer data, Shukla et al. used unsupervised data mining techniques, a self‐organizing map, and density‐based spatial clustering of applications with noise to create patient cohort clusters; they recorded the patterns linked to patient survivability and developed a deep learning model to improve the accuracy of survival prediction and determine the factors that affect the prediction [12].

A combination of the relevant clinical and genetic variables becomes a great tool for understanding the whole makeup of patient profiles [13]. Bageritz et al. concluded that there is a very high correlation between the gene expression values, thus eliminating the hardships in whole‐genome expression profiling [14]. While assessing large heterogeneous datasets to differentiate clusters using the 978 landmark genes, Clayman et al. noticed that the clusters using the landmark genes were more distinct compared to the clusters obtained using any randomly selected non‐landmark genes. They concluded that landmark genes have the ability to better represent the overall genetic profile of heterogeneous diseased tissue samples. Their clustering results varied across both the heterogeneous and the homogeneous datasets based on whether the landmark or the non‐landmark genes were used as features, and the percentage of variation captured by each of the first two principal components was greater for the landmark genes than for the non‐landmark genes [7, 9].

Omuya et al. recommended feature selection using principal component analysis (PCA) and information gain when the dimensionality poses a challenge for classification problems. They built a hybrid filter model for feature selection and concluded that the classification algorithms performed better and had reduced training time when the dimensionality was significantly reduced [15]. Jamal et al. studied the effectiveness of PCA and *k*‐means for classifying the Wisconsin breast cancer data using both SVM and XGBoost. They concluded that the *k*‐means algorithm outperformed PCA for dimensionality reduction [16]. In another study to classify the cardiotocography data, PCA was applied, and classification models built with decision trees, Naïve Bayes, random forest, and SVM demonstrated that PCA improved the performance on larger datasets [17]. Manhar et al. developed a predictive model after applying feature selection methods for heart disease classification. On the Z‐Alizadeh Sani dataset containing 54 features, Boruta, a data reduction technique based on the principles of random forest classifier, was used to select 18 features that proved to aid the classifiers in achieving higher accuracies [18]. Chen et al. also applied feature extraction using PCA in a study to detect cancer types. They concluded that dimensionality reduction using PCA is effective as a data representation method when the existing relationships are linear [19]. Rendleman et al. focused on implementing a PCA‐based dimensionality reduction and feature selection technique to reduce the model and computation complexity [20].

Dinesh et al. has proposed a predictive model to predict heart disease in humans. They have developed five models using SVM, XGBoost, random forest, Naïve Bayes, and Logistic Regression. Although all five models performed very similarly to each other, the logistic regression model reported overall better prediction accuracy [21]. In another study aimed at developing a predictive model for treating patients with temporomandibular disorders, Su et al. demonstrated the use of multinomial logistic regression (MLR) to identify potential predictors and their model showed reasonable calibration with the area under the curve (AUC) ranging between 0.76 and 0.86 [22]. Chen et al. tried to capture linear relationships in the data through PCA and then used it to predict multiple cancer types. Gene and protein networks within The Cancer Genome Atlas (TCGA) breast cancer data have also been analyzed using the random forest classifier [19]. Petralia et al., Liang et al., and Seok, all made use of feature selection, and clustering techniques to assess the impact of genes on clinical responses [23, 24, 25].

A study on drug repurposing used the *k*‐means clustering technique. Using the gene expression data, 31 disease‐type tissues were grouped into several clusters. To determine the common drugs, each cluster group was further explored to identify disease‐specific increases or decreases in the gene expression of GEO2R [26]. Wang et al. conducted an unsupervised study to identify the subtypes of major depressive disorder. They clustered the patient data based on a weighted linear combination of neurobiological and clinical features. PCA was further used to enhance and interpret their results [27]. In another study, Joel et al. used imaging data from 466 females and males to analyze human brain structure and performed anomaly detection in male and female human brains. They carried out various analytics techniques including *k*‐means and hierarchical clustering, and supervised classification with SVM and random forest. They have concluded that the human brains do not fall into one of the two distinct types of male or female brains and a human’s sex does not provide any information about typical brain architecture [28].

There have been numerous studies on the effect of genetic variables on clinical data, and various ML techniques have been used to discover those connections [29, 30, 31, 32, 33, 34, 35]. However, since profiling all the genes in the human genome requires a lot of resources, researchers came up with computational methods to profile only about 1000 carefully selected genes called “landmark genes” [7, 9, 19]. There are extensive studies being done by bioinformatics researchers to help in the identification of a particular disease’s characteristics and the underlying genetic variation patterns. This leads to identifying the risk groups and better management of the corresponding treatments [36]. Although it is believed that landmark genes have the capability to predict the expression values of the remaining genes and capture around 80% of the information of the genome, there is a need for more research to be done in this area to establish functional connections between the landmark genes and diagnosis [37, 38, 39]. There is a need to put more effort into understanding the significance and role of landmark genes in comparison to non‐landmark genes in disease‐type predictions and outcomes.

This study tries to find answers to two research questions. The first question is to find out if there is a difference in the clustering capabilities of landmark and non‐landmark genes and if the difference, if any, is statistically significant. To achieve this, samples of 10 types of diseased tissues are clustered using sets of landmark genes and non‐landmark genes, and the results are compared. The second question intends to find a smaller subset of genetic (landmark or non‐landmark) and clinical variables that can best serve as predictors for classifying disease types. For this, MLR models are constructed using both the genetic (landmark and non‐landmark) and clinical data as predictors to predict samples from 10 cancer types. Several MLR models are built using subsets of landmark and non‐landmark genes and their principal components as predictors. Their prediction accuracies are compared, and any difference is statistically tested for significance. This can be an important contribution to the field of precision medicine.

## 2 RESULTS

### 2.1 Experimental designs

#### 2.1.1 Experiments to compare the performance of the landmark genes against the different subsets of non‐landmark genes in characterizing the tissue types

The purpose of this part of the study was to compare the performance of the landmark and non‐landmark genes in characterizing 10 different diseased tissue types. More specifically, to determine if the landmark genes are better in clustering the diseased tissue types compared to the non‐landmark genes. In addition to that, the study intends to identify a subset of non‐landmark genes, if any, that performs better or the same as landmark genes in clustering the diseased tissues. Due to the dimensional complexity that is, finding a similar sized (*n* = 978) subset of genes (*n*) from the 21,290 non‐landmark genes is a combinatorial problem. There are many possibilities for coming up with a random set of 978 genes from the 21,290 non‐landmark genes. In addition to that, it is computationally impossible in a polynomial time to test all those combinations of genes to see if they have a similar or better predictive power to classify diseased tissues. Therefore, this study takes a novel approach whereby a sub‐sample of random sets of 978 non‐landmark genes is randomly selected and their predictive capabilities in classifying the diseased tissues against the landmark genes are compared. Using statistical testing, the study intends to show that a similar observation can be observed in a larger population.

Results from this study will help to understand if the clustering of diseased tissue types is better visible through the use of the landmark genes, or if any random subset of non‐landmark genes can serve as predictors to better cluster the diseased tissue types. Here it is important to note that the diseased tissue types are a proxy for the cancer types.

**FIGURE 1:**
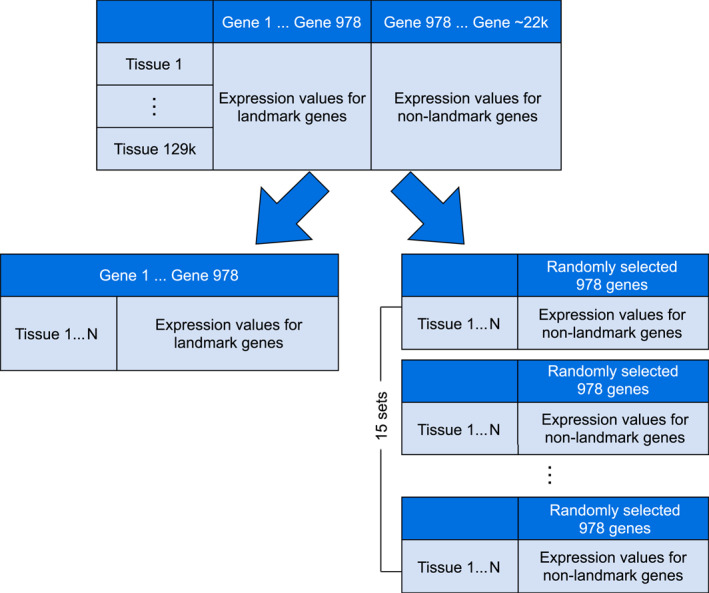
Sixteen sets of gene expression values (one set of 978 landmark genes and 15 sets of randomly selected landmark genes) were used in the study.

The gene expression data for both the landmark and the non‐landmark gene sets across the diseased tissues (L1000 dataset) were clustered using the *k*‐means algorithm. From the L1000 dataset, a total of 16 gene sets were constructed. Each gene set is composed of the expression values for 978 genes across 10 diseased tissue types. For example, in the landmark genes set (set 1), the gene expression values of the first 978 columns across 10 diseased tissue types were used for clustering. On the other hand, for each of the non‐landmark gene sets (set 2–16), a random set of 978 genes was identified within the boundary of the 979th column to the 22,678th column (see Figure 1). In addition to that, we also constructed 10 different datasets, each dataset containing a different combination of the tissue samples across 10 cancer types. A total of 160 clustering experiments using the *k*‐means algorithm were performed in this section. The 10 types of diseased tissues selected for each of the 16 gene sets were purely based on the frequency of their appearance in the dataset.

*K*‐means clustering was performed to obtain 10 clusters from each of the 16 sets using the respective genes of each set as predictors. It is important to note here that even though the classification labels of the diseased tissue types were available, unsupervised learning for classification was employed. The objective behind performing these experiments was to determine the predictive capabilities of the genes rather than to correctly classify the diseased tissues. Here, the study is interested in determining if the landmark genes can correctly classify more diseased tissues than any random subset of the non‐landmark genes. Having the knowledge of the classes (labels) for the diseased tissues is an added advantage as it can help in assigning a numerical performance measure that is, accuracy for each gene set. The gene set that records the highest accuracy in clustering the diseased tissue type can be considered to have a higher predictive capability.

**FIGURE 2:**
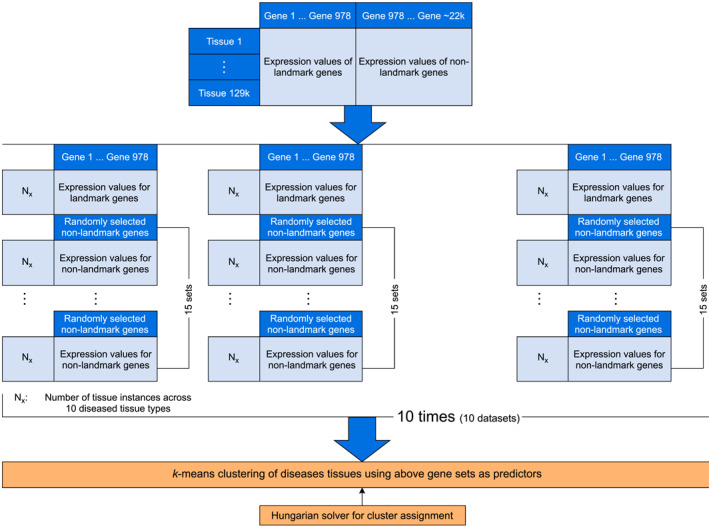
Design strategy for the *k*‐means clustering of 10 diseased tissues using 16 sets of genes.

To determine the capability of the predictors (landmark and non‐landmark genes) in clustering the instances (tissues), the clustered instances (tissues) in different clusters were compared against their known labels (tissue types) by label mapping. The label mapping was performed using the Hungarian method. The Hungarian algorithm is commonly used in assignment problems when the objective is to minimize the cost. This technique assigns the cluster labels to the tissue types by minimizing the cost using a method developed by Niclas Borlin [40]. Finally, the clustering accuracy is determined using the expression (100‐clustering error), where the clustering error is the ratio of the number of instances that were misclassified to the total number of data points. The entire experiment was repeated 10 times. Each time a total of 16 sets were constructed using a different set of 10 diseased tissue types. Across all the experiments, the genes in each set were kept intact. Hereafter, the 16 gene sets in each experiment are referred to as one dataset. Therefore, there are a total of 10 datasets. Each of the 10 datasets was independently constructed using different random seeds. It was also ensured that all the instances in the L1000 dataset had an equal probability of being selected. Figure 2 pictorially summarizes the design strategy for the experiments conducted.

#### 2.1.2 Design predictive analytics models using both the clinical and the genetic variables and predict the diseased tissue types

This study aimed to identify a smaller subset of genetic and clinical variables that can serve as predictors for classifying the diseased tissues or disease types. In the context of precision/personalized medicine, this is a very important problem to be addressed. Results from this study will allow us to understand the factors (attributes) that are essential to better understanding the onset and progression of the disease. To capture the entire landscape of the clinical and genetic variables and use it for the purpose of personalized medicine is economically expensive. There are about 21,000+ genetic variables across which the variations must be captured to profile a human being for the purpose of personalized medicine. Therefore, it becomes imperative to identify a smaller subset of the clinical and genetic variables that can serve as a profile for characterizing the disease types.

This study involves constructing MLR models using both genetic and clinical data to predict disease types. Identifying the most important set of genes and the clinical variables for predicting disease type is a dimensionality reduction problem. To reduce the complexity of the dimensions, several experiments were conducted using the feature selection techniques Boruta, boosting (XGBoost), and the dimensionality reduction technique, PCA.

During the pre‐processing stage, 16 out of 83 clinical variables were retained in the Clinical dataset as most of the clinical variables had significantly large (over 70%) missing values. From the Clinical dataset, a sample consisting of 6802 diseased tissues across 10 disease types was obtained. Both the Boruta and the XGBoost algorithms identified morphology, gender, and age at diagnosis as important clinical variables/predictors for classifying the diseased tissues. The sample dataset of dimension 6802 × 3 was further expanded to the dimension of 6802 × ∼21 k by merging it with the genetic data obtained from the L1000 dataset. The tissue identifier column in both datasets (Clinical and L1000 dataset) was used as an index to facilitate the merge.

Predictive modeling was performed on the final sample dataset using MLR. For this, the dataset was split into training and test datasets in the ratio of 70:30. The 10‐fold cross‐validation was performed on the entire dataset before the split. To handle the imbalance in the training dataset, the SMOTE‐TOMEK technique was applied. To compare the performance of different models, the 10‐fold cross‐validation accuracy on the training set and test set accuracies were reported.

### 2.2 Observations

Table 1 summarizes the classification accuracy of the 10 diseased tissue types across the 16 gene sets (one set of landmark genes and 15 sets of non‐landmark genes) for the 10 different datasets. The clustering accuracies of the non‐landmark genes across the 10 datasets (see Table 1) were averaged to represent each data point. This step was performed to obtain a set of paired data points to compare the group means of the landmark and non‐landmark genes. The Kruskal–Wallis *H*‐test, a non‐parametric equivalent of the one‐way analysis of variance (ANOVA) was performed to determine if there is a significant difference in the mean clustering accuracy of 10 diseased tissue types across the landmark and non‐landmark genes.

**TABLE 1:** Accuracies of *k*‐means clustering of diseased tissues across 10 datasets when compared with actual labels.

Dataset	LM	NLM1	NLM2	NLM3	NLM4	NLM5	NLM6	NLM7	NLM8	NLM9	NLM10	NLM11	NLM12	NLM13	NLM14	NLM15
1	0.82	0.83	0.82	0.79	0.83	0.78	0.79	0.77	0.78	0.79	0.82	0.78	0.82	0.83	0.79	0.80
2	0.90	0.78	0.81	0.79	0.89	0.86	0.92	0.85	0.85	0.53	0.84	0.75	0.83	0.89	0.66	0.79
3	0.96	0.79	0.97	0.43	0.66	0.79	0.73	0.77	0.93	0.83	0.70	0.80	0.85	0.88	0.83	1.00
4	0.86	0.80	0.67	0.94	0.84	0.77	0.90	0.79	0.85	0.88	0.55	0.86	0.94	0.79	0.84	0.97
5	0.79	0.89	0.70	0.81	0.52	0.53	0.87	0.90	0.78	0.93	0.74	0.80	0.86	0.76	0.83	0.75
6	0.92	0.96	0.88	0.84	0.91	0.89	0.88	0.63	0.77	0.70	0.91	0.78	0.72	0.65	0.82	0.81
7	0.86	0.96	0.86	0.61	0.91	0.86	0.96	0.79	0.54	1.00	0.81	0.87	0.79	0.91	0.84	0.67
8	0.75	0.87	0.57	0.84	0.85	0.79	0.79	0.68	0.74	0.94	0.88	0.83	0.87	0.96	0.82	0.76
9	0.80	0.88	0.83	0.98	0.64	0.86	0.97	0.86	0.90	0.92	0.90	0.72	0.96	0.80	0.89	0.73
10	0.80	0.87	0.91	0.66	0.90	0.96	0.87	0.63	0.89	0.69	0.79	0.86	0.86	0.94	0.88	0.84

Abbreviations: LM, landmark genes; NLM, non‐landmark genes.

At *α* = 0.1, the Kruskal–Wallis *H*‐test yielded a *p*‐value of 0.07 indicating that we can reject the null hypothesis and conclude that there is a significant difference in the clustering accuracy of the diseased tissue types across the landmark and non‐landmark genes. Post‐hoc (BH) test revealed that the landmark genes have statistically significant differences (positive) in the means compared to the non‐landmark genes. Thus, it can be concluded that the prediction capabilities of the landmark gene set in terms of clustering the diseased tissue types are slightly better than any random combination of the equally sized non‐landmark genes. The descriptive statistics of the groups (landmark and non‐landmark genes) are provided in Table 2.

**TABLE 2:** Descriptive statistics: Clustering accuracies of the landmark and non‐landmark genes across 10 sets.

Group	Count	Mean accuracy	Std deviation	Median	Interquartile range
Landmark genes	10	0.846	0.0665	0.840	0.090
Non‐landmark genes	10	0.791	0.0301	0.800	0.029

The Kruskal–Wallis *H*‐test was also performed to determine if there is a significant difference in the clustering accuracy of diseased tissue types across the 10 datasets. At *α* = 0.1, no significant difference was observed in the prediction accuracy of the diseased tissue types across the 10 datasets, as the Kruskal–Wallis *H*‐test yielded a *p*‐value of 0.3495.

#### 2.2.1 Baseline model

**FIGURE 3:**
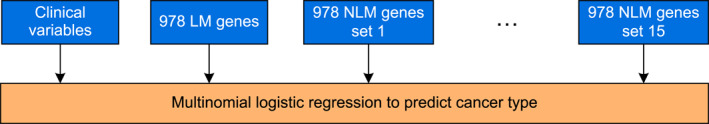
Baseline model design: Multinomial logistic regression (MLR) models with only one set of predictors.

Several models were developed to establish a baseline in this study (see Figure 3). Initially, just the three clinical variables were used as predictors to classify the 6802 diseased tissues. On the test dataset, the MLR model resulted in an accuracy of 90.48% (see Table 3). Next, the 978 Landmark (LM) genes were used as predictors.

**TABLE 3:** Test accuracies of baseline MLR models in predicting disease types using only clinical variables or landmark genes or a set of non‐landmark genes.

Model number	Predictors	Test accuracy (in %)
1	3 clinical variables	90.48
2	LM_Genes	90.74
3	NLM_set_1	85.50
4	NLM_set_2	86.04
5	NLM_set_3	85.45
6	NLM_set_4	85.20
7	NLM_set_5	85.84
8	NLM_set_6	86.77
9	NLM_set_7	86.28
10	NLM_set_8	87.51
11	NLM_set_9	85.15
12	NLM_set_10	85.30
13	NLM_set_11	86.33
14	NLM_set_12	86.62
15	NLM_set_13	86.58
16	NLM_set_14	86.87
17	NLM_set_15	85.99

Abbreviations: LM, landmark genes; MLR, multinomial logistic regression; NLM, non‐landmark genes.

The MLR model reported a slightly better accuracy (90.74%) when compared to just using the three clinical variables. Finally, all 15 sets of non‐landmark genes (NLM set x) were used as predictors. The test accuracies of the MLR models using the different sets of NLM genes as predictors are recorded in Table 3. Overall, the landmark genes outperformed all the 15 different sets of randomly selected 978 NLM genes (mean accuracy of NLM genes is 86.1%) in classifying the 6802 diseased tissues across 10 disease types.

#### 2.2.2 Model improvement

**FIGURE 4:**
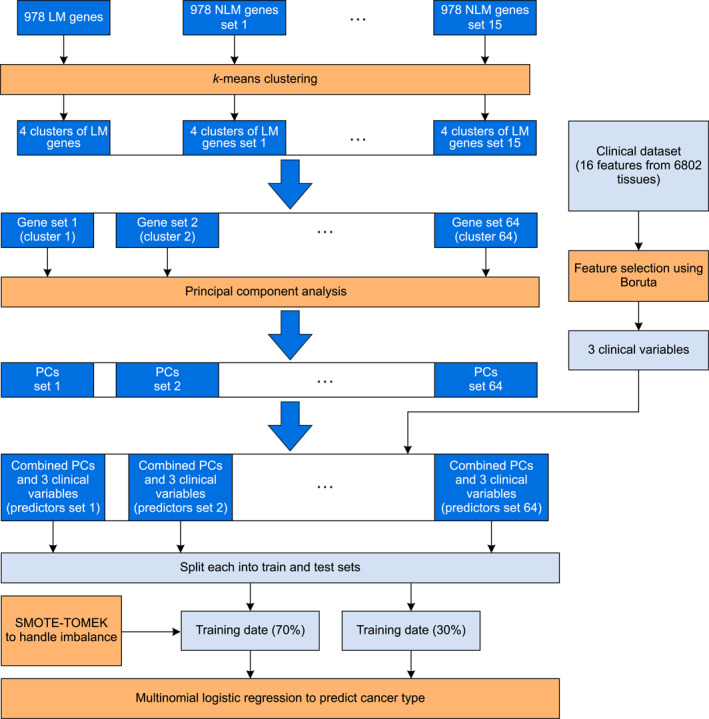
Experimental design strategy for model improvement by applying *k*‐means and principal component analysis (PCA) of the predictor variables.

With the baseline established, the next objective was to determine if the predictive capabilities of MLR can be boosted when both the clinical and the genetic variables are used together as predictors. In this section, a total of 64 experiments were designed. To begin with, *k*‐means clustering was performed on the 16 sets of genetic variables (one set of LM genes and 15 sets of NLM gene sets). Each gene set was clustered into four clusters based on the “Elbow method”. A total of 64 gene clusters were obtained. To further reduce the dimensions of the gene clusters, PCA was performed (see Figure 4). Across each gene cluster, only those principal components (PC) that resulted in an eigenvalue greater than 1 were selected for further analysis.

Table 4 shows the 10‐fold cross‐validation training and test accuracies for the diseased tissue type predictions by the MLR classifier using different sets of predictors. It is important to note that the three clinical variables were used consistently as predictors in all 64 experiments. However, in each of the experiments the PCs, a representation of the linear combination of the original genetic variables, were used to replace the genetic variables. For example, in the first experiment, 128 PCs were obtained from the genes in one of the four clusters of landmark genes and the three clinical variables were used as predictors to classify the diseased tissues. Table 4 also records the total number of PCs used across each cluster as a representative of the genetic variables for the MLR models (see column “# PCs”). Based on the training and test set accuracies reported in Table 4 we did not observe any overfitting of the MLR model.

**TABLE 4:** Prediction results of multinomial logistic regression (MLR) models when principal components (PCs) are added to the three clinical variables: training and test accuracies are shown in percentages.

Cluster name	# PCs	Train	Test	Cluster name	# PCs	Train	Test
lm_c1	128	96.4	96.63	nlm_c2_7	295	97.6	96.78
lm_c2	130	97.5	97.12	nlm_c2_11	316	93.1	94.30
lm_c3	157	97.1	97.32	nlm_c2_3	141	93.0	94.00
lm_c4	61	97.2	96.73	nlm_c4_5	59	97.3	96.68
nlm_c1_14	318	96.6	94.40	nlm_c4_4	23	98.5	94.35
nlm_c3_11	70	95.9	96.73	nlm_c2_2	25	97.4	96.73
nlm_c3_10	19	97.8	96.83	nlm_c2_14	72	92.1	94.49
nlm_c3_12	131	96.3	96.53	nlm_c2_0	75	96.8	96.28
nlm_c3_13	18	97.8	95.14	nlm_c4_6	129	96.2	96.68
nlm_c1_12	323	98.0	94.49	nlm_c4_7	25	98.1	93.95
nlm_c3_9	297	95.0	93.95	nlm_c2_1	58	96.8	95.88
nlm_c3_8	24	96.2	96.13	nlm_c4_11	123	97.1	96.38
nlm_c1_13	67	98.2	94.59	nlm_c3_6	71	96.6	96.97
nlm_c1_11	30	97.6	96.28	nlm_c1_4	56	97.4	96.33
nlm_c3_14	123	93.1	94.30	nlm_c1_5	318	92.2	93.55
nlm_c1_8	309	91.3	94.25	nlm_c4_10	61	92.3	93.75
nlm_c1_9	139	93.3	94.44	nlm_c3_7	138	98.1	94.64
nlm_c1_10	320	97.8	96.48	nlm_c3_5	136	98.2	94.05
nlm_c4_9	68	96.2	95.83	nlm_c4_12	22	93.9	94.79
nlm_c4_8	75	97.9	96.43	nlm_c1_7	74	96.9	96.73
nlm_c2_9	26	97.7	96.43	nlm_c1_6	308	93.5	94.40
nlm_c2_8	133	98.8	94.94	nlm_c3_4	139	97.5	96.73
nlm_c2_13	332	97.8	96.38	nlm_c4_13	128	97.7	96.68
nlm_c2_5	24	96.4	95.78	nlm_c3_0	25	97.9	96.03
nlm_c4_3	66	92.1	93.65	nlm_c1_2	131	98.4	94.89
nlm_c4_2	71	96.8	96.18	nlm_c1_3	312	92.2	94.15
nlm_c2_4	304	97.9	94.54	nlm_c3_1	25	96.2	96.18
nlm_c2_12	59	98.0	94.54	nlm_c3_3	23	95.8	94.20
nlm_c2_10	143	98.1	94.35	nlm_c4_14	31	97.9	94.40
nlm_c2_6	32	98.8	94.69	nlm_c1_1	323	98.5	97.12
nlm_c4_0	130	98.6	93.90	nlm_c1_0	314	96.2	94.49
nlm_c4_1	133	93.3	94.20	nlm_c3_2	303	95.3	94.69

## 3 DISCUSSIONS

One‐way ANOVA is performed to determine if there is a significant difference in the test accuracies of the MLR models using a different set of predictors (PCs representing the clusters of the genetic variables). At a significance level of *α* = 0.1, the *p*‐value obtained was 0.06. Therefore, we reject the null hypothesis and conclude that there is a significant difference in the accuracies of the models when using different clusters of genetic variables as predictors in the MLR classification. Using the Post‐hoc (Tukey’s) test revealed that the PCs that are representatives of the LM clusters have statistically significant differences (positive) in the means compared to the PCs that are representatives of the NLM clusters. The MLR classification models, with the landmark genes as predictors slightly outperformed the non‐landmark genes as predictors in classifying the diseased tissue across 10 different cancer types.

**FIGURE 5:**
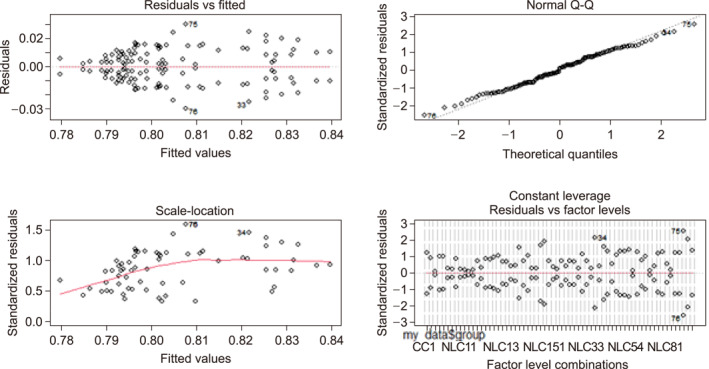
Plots to check for normality assumptions.

The residuals versus fitted plot shown in Figure 5 shows the ﬁtted values plotted against the model residuals. The ﬂat red line looks very good indicating that the single predictor variable is sufficient to explain the dependent variable, that is, the accuracies of the classification. The normal Q‐Q plot in Figure 5, shows the quantiles of the standardized residuals plotted against the quantiles. Since the standardized residuals fall mostly on the straight line, the assumption of normally distributed residuals is met. In addition to the normal Q‐Q plot, the Shapiro–Wilk normality test was also performed to confirm the non‐validity of the normality assumption. At *α* = 0.1, the Shapiro–Wilk normality test resulted in a *p*‐value of 0.6 suggesting not to reject the null hypothesis indicating that the samples come from the normal population. The scale‐location plot in Figure 5, shows the square root of the absolute standardized residuals plotted against the ﬁtted or predicted values. Since the red line that ﬁts the standardized residuals is somewhat ﬂat, it indicates that the spread in the predictions is almost the same across the prediction line, indicating a very low chance of the failure of the homogeneity of variance across the different clusters of the genetic variables. Finally, the residuals versus leverage plot in Figure 5, shows a measure of the influence of each point on the overall equation against the standardized residuals. Since no points stand out far from the line, we can assume that there are no outliers having undue influence on the ﬁt of the model.

To summarize, this study primarily demonstrates the promise of the MLR classifier models, and the ability of the latent genetic variables (using principal components) to predict the disease types. It is worth noting that the genetic variables or their latent representations can serve as a proxy for the clinical variables.

## 4 CONCLUSIONS

The objective of this study was to understand the differences in functionality, including the predictive capabilities of the landmark and non‐landmark genes. Using both supervised and unsupervised techniques, the study tried to cluster and classify the diseased tissue samples. Both the genetic and the clinical variables, as predictors, have been explored for classification purposes. More specifically, this study aimed at addressing the following research questions: (1) Is there a significant difference in the performance of the landmark and non‐landmark genes when used for clustering different types of diseased tissues, and (2) to identify a smaller subset of genetic and clinical variables that can serve as predictors for classifying the diseased tissues or disease types.

Several studies in the past have only focused on the abilities of the expression values of the landmark genes to derive the expression values of the non‐landmark genes but have not discussed if the landmark genes have superior predictive capabilities compared to the non‐landmark genes in terms of clustering/classifying the diseased tissue types. Experiments performed in this study indicate that the set of landmark genes has slightly better (statistically significant) predictive capabilities in clustering/classifying the diseased tissue types when compared to any randomly chosen sets of non‐landmark genes. Various experiments to predict the diseased tissue types showed that both the clinical and the genomic variables are important predictors. Our model demonstrated a significant increase in prediction capabilities when compared to the baseline model in which either the clinical or the genetic variables alone were used as predictors. It was found that the clinical variables namely morphology, gender, and age at diagnosis, serve as the top three predictors for predicting the diseased tissue types. However, some of the clinical features such as morphology is only associated with the manifestations of the disease and are a measurement that is captured after the manifestation of the disease. Therefore, identifying a subset of genetic variables that can serve as relevant clinical biomarkers or proxies for the clinical variables becomes vital in the field of precision medicine. To reach that goal, this study tried to identify a subset of genes. By employing ML techniques, different clusters of the genetic variables were obtained, and their derived latent representations were used as predictors for the classifier. These experiments demonstrated that the principal components of the clusters of the landmark genes have slightly better (statistically significant) predictive capabilities in classifying the diseased tissues when compared to any set of principal components obtained from the clusters of the non‐landmark genes. Thus, it is evident that the landmark genes have the capability to serve as a proxy for the clinical variables.

Precision medicine or personalized medicine is ever focused on identifying specific genes that contribute to a disease and therefore can represent clinically relevant biomarkers for that disease. Hence, the findings from this study are relevant to this field. The fact that clinical variables are measured only after the manifestation of the disease makes this kind of study that can spot genetic contributors, critical in the field of precision medicine. Moreover, the need to identify precisely the genetic and clinical contributors to disease has been fueled by the large‐scale availability of gene expression profiling and clinical data being gathered from cancer datasets. While clinical features of a disease help in understanding its onset and progression, the genetic features that lead to the disease serve as biomarkers to identify genetic predisposition or genetic susceptibility. Even though environment and lifestyle are also contributing factors to the disease manifestation, knowledge of genetic makeup is key to precision/personalized medicine.

## 5 MATERIALS AND METHODS

### 5.1 Dataset

A total of two datasets were used in this study—the clinical data and the microarray version of the LINCS L1000 dataset. Clinical data consists of clinical and genetic information for tissues across 55 cancer types. This comes from The Cancer Genome Atlas (TCGA), a cancer genomics program that has compiled a comprehensive set of datasets through the National Institute of Health. Since its start in 2006, TCGA has accumulated over 2.5 petabytes of genomic, epigenomic, transcriptomic, and proteomic data and has paved the way for progress in our ability to diagnose, treat, and prevent cancer through various applications including predictive analytics and ML. The National Cancer Institute’s Genomic Data Commons (GDC) is a data repository that hosts the TCGA data along with other cancer research data and was started in June 2016 for its applications in precision medicine and oncology. The GDC data portal permits the querying and downloading of raw and processed data in addition to providing exploration and visualization tools.

This study took a total of 13,122 observations (tissues) across 83 clinical and 22,268 genetic variables. L1000 data molecularly characterizes over 120,000 primary cancer and matched normal tissue samples stretching across 33 cancer types. Then a subset of 6802 diseased tissues from 13,122 observations in clinical data across 10 cancer types was used. For all the 6802 observations a profile was created using 22,268 genetic variables and only 16 clinical variables. Out of the 16 clinical variables, 3 clinical variables namely morphology, age at diagnosis, and gender were identified as important variables using the Boruta feature selection tool. In addition, a strong correlation between these three clinical variables with the cancer type was observed. A sample of the reduced Clinical dataset is shown in Table 5.

**TABLE 5:** A snapshot of the Clinical dataset showing the three most important variables morphology, age at diagnosis (in days), and gender.

Morphology	Age at diagnosis	Gender	Cancer type
8500/3	17,689	Female	Breast
8120/3	23,116	Female	Bladder
9401/3	11,509	Male	Brain
8140/3	21,748	Male	Prostate gland
8140/3	24,030	Male	Prostate gland
8144/3	20,941	Male	Stomach
8120/3	32,872	Male	Bladder
8500/3	18,942	Female	Breast
8310/3	26,060	Male	Kidney
8520/3	32,872	Female	Breast

**FIGURE 6:**
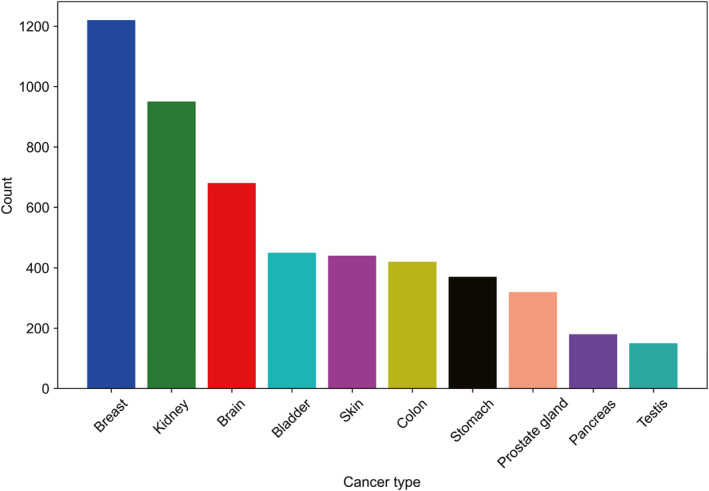
Distribution of 10 different cancer types across 6802 tissue samples.

The distribution of the observations across 10 cancer types is shown in Figure 6. Due to the high dimensionality of the datasets, the descriptions of the individual predictors are not provided here. In addition to that, it was noted that most of the clinical variables contained missing values. The pre‐processing of the clinical data involved the removal of any clinical variables with rows that contained more than 70% missing values.

The L1000 data, comprising 978 landmark genes and 21,290 non‐landmark genes, is approximately 10.7 GB in size and the values of each cell in the matrix were quantile normalized to range between 4 and 15. This file was available for download in the Gene Cluster Text format, a binary file format used to store annotated data matrices [19, 41].

The CMapPy, a Python module was used to read and analyze the file. In the L1000 dataset, the tissues are referenced using the GSM_ID which was used as tokens for retrieving additional details about the tissue from the GeoQuery database using the GeoQuery library. All the GSM_IDs were replaced by the tissue types that is, by their name.

### 5.2 Tools and techniques

This section discusses all the tools and techniques employed in this study. Both the supervised and unsupervised classification techniques were used in this study. The clustering algorithm, *k*‐means was used to cluster similar tissue types. However, the objective was to determine a subset of the clinical and genetic variables that can distinctly cluster similar tissue types. For the experiments that required supervised classification, MLR was preferred and Boruta, a feature selection tool, was used to select clinical variables.

#### 5.2.1 *k*‐means clustering

Cluster analysis or clustering is an unsupervised ML technique that identifies natural data groups or clusters by evaluating individual data points and assigning each one of the data points to a group (cluster). Applications of cluster analysis include medical image processing, bioinformatics, anomaly detection, and many more. There are various types of clustering techniques such as hierarchical clustering (connectivity‐based), *k*‐means clustering (centroid‐based), distribution‐based, and density‐based to name a few [42, 43, 44].

Centroid‐based clustering is one of the most effective methods of clustering while still being the simplest and the fastest for large datasets. This method of clustering can be applied to numerical data only. The *k*‐means algorithm partitions *N* data points within a vector space into *K* distinct clusters. The data points are allocated to the closest cluster, and the clusters evolve to fit the data naturally. It works by minimizing the intra‐cluster variance where the defined clusters minimize the sum of the squared distances between data points and the center of the cluster they are part of. The algorithm needs the user to specify *K*, the number of clusters before it starts, either intuitively or by using the Elbow method. The center of a cluster is calculated by repeatedly assigning points to the closest centroid and updating them using the Euclidean distance from the centroid to the data points until the stopping criteria (either the algorithm converges, or the maximum number of iterations has reached) [42, 45]. The Euclidean distance (square‐norm) is the cost function used.

#### 5.2.2 Multinomial logistic regression

MLR is an extension of binary logistic regression because it has more than two categories of the target variable. The independent variables can be categorical or continuous. MLR models estimate the relationship between a set of predictors and a multicategory nominal (unordered) target variable without making any assumptions. Instead of modeling the outcome directly, the method models the log odds as a linear combination of the predictor variables using the logistic function. Therefore, it uses the maximum likelihood estimation to determine the probability of the dependent variable [42].

#### 5.2.3 Principal component analysis for dimensionality reduction

PCA is an unsupervised ML algorithm that is used for applications such as dimensionality reduction, exploratory data analysis, information compression, and data de‐noising to name a few. The algorithm finds new axes (orthogonal projections of data) that preserve a higher variance for data in lower dimensions. These axes are called PC. PCA is highly sensitive to the scale of the features. Therefore, the continuous predictors are standardized first, then the covariance matrix is computed, and finally, eigenvalues and eigenvectors are computed [42, 46]. Mathematically, if the original input numeric variables are *X*_1_,*X*_2_,⋯,*X*_*n*_ then *PC*_*i*_ = *w*_*i*1_ *X*_1_ + *w*_*i*2_ *X*_2_ + ⋯ + *w*_*in*_ *X*_*n*_, where *w*_*ij*_ is a component loading between *PC*_*i*_ and *X*_*j*_, for 1 ≤ *i*,*j* ≤ *n*.

Eigenvectors give the direction in which the data is spread out and eigenvalues give their relative importance. Since the number of principal components given by PCA is equal to the number of features, eigenvalues can be used to decide the number of principal components to retain. A PC with an eigenvalue greater than one implies that it accounts for more variance than one of the original features in the standardized data and therefore an eigenvalue greater than one can be chosen as a criterion for retaining a PC [42, 46]. A limitation of PCA is that it assumes linear relationships among features.

#### 5.2.4 Boruta for feature selection

Feature selection is the process of reducing the number so that the computational costs are reduced which in turn sometimes improves the performance of the model. Boruta is one such feature selection algorithm that works as a wrapper algorithm around random forest. It follows an all‐relevant feature selection method where it captures all features that are relevant to the target variable. This is important because removing a redundant variable most certainly improves a model’s accuracy. Also, too many variables might result in overfitting [18, 47].

The Boruta algorithm starts with shuffling the values of all the independent features and merges them with the original features. It then trains a random forest model on the new dataset functions by performing the shuffling of predictors’ values and joining them with the original predictors and then by building a random forest on the merged dataset. Then, it calculates the importance of the original variables by comparing them with its randomized dataset and chooses all the predictors with higher importance. The advantages of using Boruta as a feature selection technique are many. It employs a strategy of including all the predictors to start with and then eliminating them based on their statistical importance in a top‐down approach. It handles interactions between the predictors and their multi‐variable relationships and can be used to develop classification and regression models [18, 47]. Boruta serves as an important feature selection tool for biomedical applications.

#### 5.2.5 The Hungarian algorithm for cluster assignment

The Hungarian algorithm also known as the Munkres Assignment algorithm is a combinatorial optimization algorithm used to solve linear assignment problems. Though it is a classical method for solving assignment problems, it can be used in matching problems such as matching labels in clustering. The algorithm can be divided into several steps—the first step is to reduce the matrix, the next step is to cross zeroes, and finally reduce again until the elements can be paired [40].

#### 5.2.6 SMOTE‐TOMEK technique for handling class imbalance in the data

Generally, imbalanced data can be handled using one of the two techniques: oversampling and undersampling. Oversampling techniques synthesize new examples of the minority classes so that they match the number of majority classes. Synthetic minority over‐sampling technique (SMOTE), adaptive synthetic, and Random Over Sampling are some of the oversampling techniques. The disadvantage of oversampling is that by generating multiple samples within the minority class, overfitting is highly likely. Additionally, the high dimensionality of the data poses a problem because SMOTE generates synthetic examples but does not take into consideration the neighboring examples from other classes. This results in an increase in the overlapping of classes and can introduce additional noise. Undersampling techniques, on the other hand, remove majority class data to match the number of minority classes. TOMEK links, ENN, and Random Under Sampling are some of the undersampling techniques. However, one disadvantage of undersampling techniques is that the data points belonging to the majority class are lost [48].

To overcome the disadvantages of oversampling and undersampling techniques, the oversampling technique can be combined with an undersampling technique, and the resulting hybrid technique can better handle the imbalance in the dataset. SMOTE or synthetic minority oversampling technique can be combined with TOMEK links, an under‐sampling technique to optimize the performance of the models [49]. TOMEK links are the opposite class paired samples that are the closest neighbors to each other. This technique works by removing the majority class data points wherever there are pairs of data points belonging to different classes. Therefore, TOMEK increases the class separation and decreases the number of majority class samples. To combine SMOTE with TOMEK, the first SMOTE is applied to create new synthetic minority samples to balance the data set. Then, TOMEK links are used to remove the data points close to the boundary of the two classes. As a result of the SMOTE‐TOMEK technique, the separation between the two classes is increased, thereby reducing noise.

#### 5.2.7 Stratified sampling

Stratified sampling is performed to ensure that each characteristic in a population is well represented when a population’s characteristics are diverse. It forces the distribution of the target variable among different splits to be the same. The total population is divided into homogeneous subpopulations called strata. The strata are then used to randomly select the sample population [50]. In other words, even the smallest subgroup gets represented. It not only provides better coverage of the population but also, by applying stratified sampling, training is performed on the same population, in which it is being evaluated, achieving better performance. Without stratified sampling, training and evaluating done in heterogeneous groups would result in prediction errors.

#### 5.2.8 Statistical tests

##### Analysis of variance

ANOVA is a commonly used, yet extremely powerful statistical technique to compare two or more populations of interval data. The ANOVA technique determines whether differences exist between population means. The technique analyzes the sample variances (thus the name, ANOVA) to determine if the population means differ. When the procedure is applied to independently drawn samples, it is called one‐way ANOVA [50]. This test assumes independence of observations, a normally distributed dependent variable, and homogeneity of variance.

##### Kruskal–Wallis *H*‐test

It is a statistical test to compare two or more populations when the data is either ordinal or interval, but non‐normal and the samples are independent. It is a nonparametric counterpart of one‐way ANOVA. Since the data are ordinal or are treated as ordinal, the population locations are tested instead of population means.

## AUTHOR CONTRIBUTIONS

**Madhuri Gollapalli**: Conceptualization; data curation; formal analysis; investigation; methodology; software; visualization; writing—original draft. **Harsh Anand**: Conceptualization; formal analysis; methodology; software; visualization; writing—review & editing. **Satish Mahadevan Srinivasan**: Conceptualization; data curation; methodology; project administration; supervision; resources; validation; writing—review & editing.

## CONFLICT OF INTEREST STATEMENT

The authors Madhuri Gollapalli, Harsh Anand, and Satish Mahadevan Srinivasan declare that they have no known competing financial, professional, or personal relationships that could have appeared to influence the work reported in this paper.

## ETHICS STATEMENT

This article does not involve any studies with human or animal subjects conducted by any of the authors.

## Data Availability

All the Python scripts employed in this study to perform the experiments reported in this study can be obtained from the GitHub repository.
